# An Automatic Stationary Water Color Parameters Observation System for Shallow Waters: Designment and Applications

**DOI:** 10.3390/s19204360

**Published:** 2019-10-09

**Authors:** Wenkai Li, Liqiao Tian, Shanshan Guo, Jian Li, Zhaohua Sun, Li Zhang

**Affiliations:** 1 State Key Laboratory of Information Engineering in Surveying, Mapping and Remote Sensing, Wuhan University, Wuhan 430079, China; liwenkai@whu.edu.cn (W.L.); tianliqiao@whu.edu.cn (L.T.); zhanglinu@126.com (L.Z.); 2 The Second Design and Research Institute of Water Conservancy Hydropower of Hebei Province, Shijiazhuang 50021, China; whuguoshanshan@163.com; 3 School of Remote Sensing and Information Engineering, Wuhan University, Wuhan 430079, China; 4 CAS Key Laboratory of Ocean and Marginal Sea Geology, South China Sea Institute of Oceanology, Chinese Academy of Sciences, Guangzhou 510301, China; Joeysun@scsio.ac.cn

**Keywords:** Water-leaving reflectance, above-water method, automatic sun-tracking platform, water color parameters, wireless transmission

## Abstract

Measurements of the above-water spectrum and concerned water color parameters (WCPs) are crucial for research and applications in water environment remote sensing. Due to the lack of system integration and automatization, conventional methods are labor-intensive, time-consuming, and prone to subjective influences. To obtain a highly accurate and long-term consistent spectrum and concurrent WCPs (Chl-a (chlorophyll-a), turbidity, and CDOM (Colored Dissolved Organic Matter)) data with a relatively low cost, an Automatic Stationary Water Color Parameters Observation System (AFWCPOS) was developed. Controlled by an automatic platform, the spectral and WCPs data were collected by TriOS RAMSES hyperspectral spectroradiometers and WETLabs ECO (Environmental Characterization Optics) fluorometers following the measurement protocol. Experiment and initial validations of AFWCPOS were carried out in Poyang Lake, the largest freshwater lake in China, from 20 to 28 July 2013. Results proved that the spectral data from AFWCPOS were highly consistent with the commonly used portable SVC (Spectra Vista Corporation) HR-1024 field spectroradiometer, with the coefficient of determination (*R*^2^) of 0.96, unbiased percent difference (UPD) of 0.14, and mean relative difference (MRD) of 0.078. With advantages of continuous and high degrees of automation monitoring, the AFWCPOS has great potential in capture diurnal and inter-diurnal variations in the test site of Poyang Lake, as well as another high-dynamic shallow coastal and inland waters, which will benefit routine water quality monitoring with high quality and high-frequency time-series observations. In addition, a successful case based on Landsat 8 OLI (Operational Land Imager) image and in-situ data collected by AFWCPOS showed it’s potential in remote sensing applications. The spatial distribution of Chl-a, turbidity, and CDOM were mapped, which were explainable and similar to previous researches. These results showed our system was able to obtain reliable and valuable data for water environment monitoring.

## 1. Introduction

Dramatic changes have occurred in lakes driven by climate change and human activities in the past several decades [1,2,3,4]. As habitats of aquatic organisms, lake ecosystems are facing serious pollution problems [5,6]. To monitor, protect, and repair the ecosystem, many water quality parameters (e.g., turbidity and chlorophyll-a) were measured as indicators to assess the level of lake ecosystem health. Satellite ocean color remote sensing is an important technology of obtaining crucial water variables [7,8]. The remote sensing reflectance *Rrs* determined from the top-of-atmosphere radiance is fundamental for higher-level products, such as chlorophyll-a (Chl-a), total suspended sediments (TSS), and Colored Dissolved Organic Matter (CDOM) concentration. Therefore, accurate in-situ reflectance measurements are important for modeling and validations of the satellite remote sensing products [9]. In addition, due to cloud coverage, weak light, and other reasons, optical remote sensing is incapable of observing highly dynamic water most of the time; in-situ measuring can overcome these problems as a method of water monitoring.

In general, spectrum and water color parameters (WCPs) are acquired from in-situ measurements. Above-water and in-water spectral measurement methods have been commonly used for in-situ water-leaving reflectance collection. The in-water method relies on continuous or discrete measurements along the water profiles. Accurate determination of the *Rrs* from a profile requires deep water to minimize the effects of waves during the depth extrapolation, and are frequently affected by bottom properties and vertical gradients of optically significant constituents just below the surface [10]. Therefore, the in-water method is relatively difficult and seldom adopted in shallow inland waters. The above-water method is suitable for in-situ spectra collection for inland shallow waters, and the *Rrs* is determined by subtracting the sky radiance *Lsky* from the total radiance Lu. Measurements of the *Lsky* are carried out in the same azimuthally plane as the *L*u, but at a different viewing angle (i.e., equivalent to a zenith angle) [9]. However, accurate above-water *Rrs* acquisition is challenging because all measurements (*Lu*, *Lsky*, *Ed*) should be simultaneously made using the recommended viewing geometry to avoid influences of instruments and platform shadow and to minimize sky and sun glint, as well as the reflection from the ocean surface [11,12,13]. In addition, all measurements are made based on the assumption that the downwelling irradiance (Ed) is stable during the data collection and can be measured from a standard plaque.

Automatic measurements without manual intervention can help to ensure the data quality of the optical measurements. In recent years, some multi-channel spectral systems have been developed with fixed view geometry, including WISP-3 (Water Insight Spectrometer), TriOS, and HySAS, etc. WISP-3 is a hand-held radiometer which automatically performs measurements with three radiometers (*Lsky*, *Lu*, and *Ed*) and does not need to be connected with cables during measurements [14]. The TriOS radiometric measurement system consists of three TriOS-RAMSES hyperspectral radiometers (350 to 950 nm). The radiometers can be deployed above or below the water surface. The Satlantic HyperSAS remote sensing system is designed for above-water measurements of ocean color using the Satlantics OCR-3000 (MiniSpec) series of digital optical sensors, which work similarly as the other two systems [15]. To enhance the level of automatization further, some systems tracking the sun and correct viewing geometry have been developed for minimizing the uncertainty of manual operation. The RFlex system can be used for autonomous reflectance measurements with GPS signal parsing, controlling the viewing angle of the sensor platform (https://sourceforge.net/projects/rflex/). The SeaBird Scientific developed a similar system called SAS (Surface Acquisition System) Solar Tracker (http://www.seabird.com/solartracker). Ships with the RFlex system or SAS Solar Tracker both have been operating on the different projects [16]. Due to the lack of integrated data logging and transmission hardware unit or power unit, this system cannot be used as an unattended stationary system in long-term filed measurements.

In terms of WCPs, sampling, filtration, drying, weighing, or measurement using spectrometers are key steps for laboratory analysis. The lab-measuring method demands huge finance, personnel investments, and cannot be used for continuous observations. It is effective and accurate to measure WCPs by optical sensors of in situ measurements. Some equipment, including microFlu fluorometer, WETLabs Environmental Characterization Optics (ECO) series fluorometers, YSI 660 sonde, and Sequoia Scientific LISST sensor, can be deployed to capture continuous and concurrent WCPs measurements.

Although spectrum and WCPs could be measured by the above-mentioned instruments, there is still a lack of an integrated system to automatically collect and broadcast data, which is important for fixed observation station. The goal of this study was to develop an unmanned observation system to obtain time series, highly frequent, and highly accurate spectrum and WCPs continuously for water environment monitoring. Therefore, an Automatic Stationary Water Color Parameters Observation System (AFWCPOS) based on spectral collection unit, WCPs measurement unit, and wireless transmission unit was proposed. Testing and applications of the system were carried out in Poyang Lake, the largest freshwater lake in China. Cross-comparison and validation of the spectrum were achieved using synchronous data determined from the SVC (Spectra Vista Corporation) HR-1024 field-portable spectroradiometer, and dynamic features were analyzed using Chl-a, turbidity, and CDOM concentration data collected by WETLabs ECO fluorometers. Then, we built WCPs retrieval algorithms based on these test data and mapped spatial distributions of Chl-a, turbidity, and CDOM from Landsat 8 OLI (Operational Land Imager) image.

## 2. Theory and Approach

### 2.1. Spectrum Measurement and Automatic Sun-Tracking

The remote sensing reflectance at wavelength *λ* just above the water, *R_rs_*(*λ*) (sr^−^^1^), is defined as the ratio of the water-leaving radiance *L_w_*(*λ*) (mW·cm^−^^2^·μm^−^^1^·sr^−^^1^) originating from the nadir to the downward irradiance *E_d_*(*0^+^,λ*) (mW·cm^−^^2^·μm^−^^1^) above the surface. To determine *L_w_*(*λ*), the total above-water radiance *L_u_*(*λ*) must be corrected for the skylight reflection (sky and sun glint), *L_sky_*(*λ*), which is directly reflected by the air-water interface [6,7,8]:

(1) $$R_{rs}=\frac{L_{W}\left(\lambda \right)}{E_{d}\left(0+,\lambda \right)}=\frac{\left(L_{u}\left(\lambda \right)-\rho_{f}L_{sky}\left(\lambda \right)\right)}{E_{d}\left(0+,\lambda \right)}$$

where *ρ_f_* is the reflectance factor of rough water surface. The WISP-3, TriOS, and HySAS systems measure the sky radiance, *L_sky_*(*λ*), and the total upwelling radiance underwater, *L_u_*(*λ*). A separate sensor is used to measure the downwelling irradiance, *E_d_*(*0^+^,λ*). If *E_d_*(*0^+^,λ*) cannot be measured directly using an irradiance sensor, a single radiance sensor is used, and *R*_rs_(λ) is calculated using the following equation:

(2) $$R_{rs}=\frac{L_{W}\left(\lambda \right)}{E_{d}\left(0+,\lambda \right)}=\frac{\rho_{plaque}\left(L_{u}\left(\lambda \right)-\rho_{f}L_{sky}\left(\lambda \right)\right)}{\pi L_{plaque}\left(\lambda \right)}$$

where $L_{plaque}\left(\lambda \right)$ is the reflective radiance of a standard reference plaque, and $\rho_{plaque}$ is the reflective ratio of standard reference plaque, which is an inherent property and calibrated by lab-measuring. Single-channel spectroradiometer, such as Optics HR2000, ASD Field Spec4, SVC HR 1024, and SE PSR 3500 work in this way.

**Figure 1:**
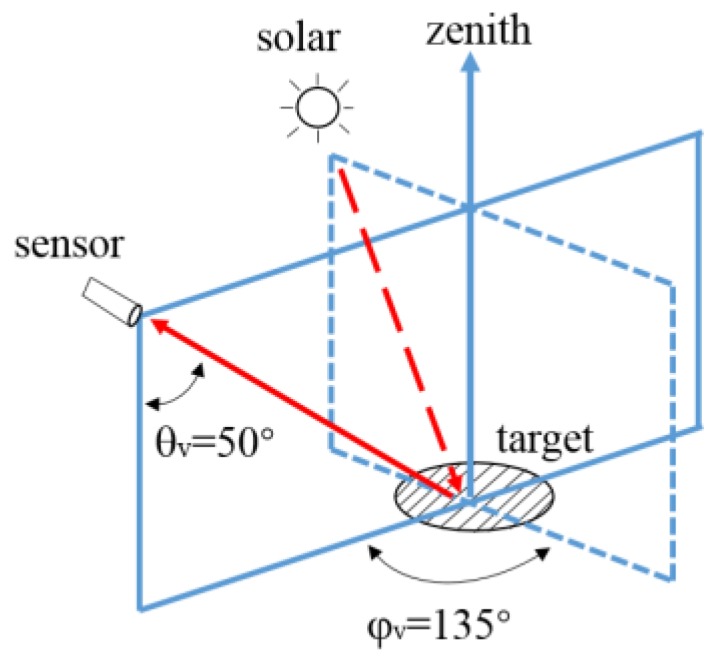
The viewing geometry of 50° from nadir (θ_v_) and 135° from the sun (φ_v_) in the above-water method [13].

**Figure 2:**
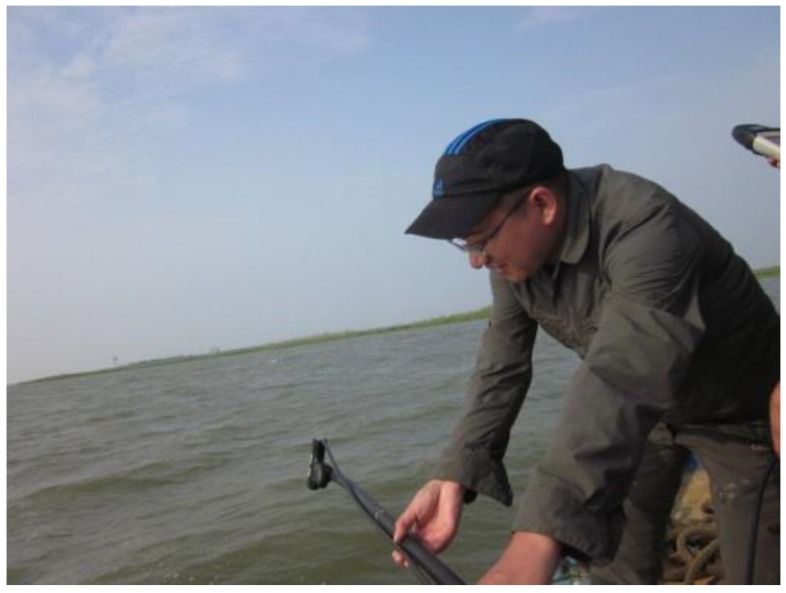
The uncertainties in viewing geometry with manual operation.

In order to reduce the influence of direct solar reflection and ship shadow on the Light Field, the recommended viewing geometry must be implemented (Figure 1). The combination of φ_v_ = 135° from sun and θ_v_ = 50° from nadir can minimize uncertainty, which is impractical for routine monitoring, and it is an error-prone task associated with manual operation (Figure 2).

Accurate determination of *R*_rs_(λ) mainly depends on the ability to minimize the glint following a standard viewing geometry [13] and applying statistical filtering schemes to *L*_u_(λ) [9,12]. Some research is applied physically based on the correction methods that rely on prior knowledge of the optical properties of water in the near-infrared spectral region [17] or by using polarization to directly reduce the sky- and sun-glint [11]. Instead of manual measurements made from ships or boats, the automatic remote-controlled measuring system, the AFWCPOS, is deployed in a fixed platform. With an automatic sun-tracking platform (ASTP) proposed in this research, all measurements and viewing geometries could be performed without any intervention. Using the sun-tracking platform, the solar azimuth angle, which defines the solar direction, is calculated through a set of successive steps based on the site location and measurement time [18].

### 2.2. WCPs Measurement

The lab-measuring cannot be used for the unattended measurement system, and, therefore, one optical WCPs measurement unit is necessary. In this study, three WETLabs ECO fluorometers were installed to AFWCPOS as an underwater unit, and they could work all weather and time for obtaining CDOM, Chl-a, and turbidity data. The WETLabs ECO fluorometers are capable of monitoring water constituents’ concentrations online by directly measuring the amount of fluorescence emission from a given sample volume. The ECO-FLNTU sensor is sensitive in optical scattering measurement at 700 nm for determining turbidity and is not affected by CDOM concentration. For the ECO-FL sensor, the Chl-a is excited by an external light source at 470 nm and re-emits a small portion of the absorbed energy as fluorescence at a longer wavelength at 695 nm. While CDOM is excited at 370 nm and re-emits at 680 nm for ECO-FLCD. The instrument-specific calibrations were performed using WETLabs supplied scale factor, dark counts, etc.

## 3. Design and Implementation of the AFWCPOS

**Figure 3:**
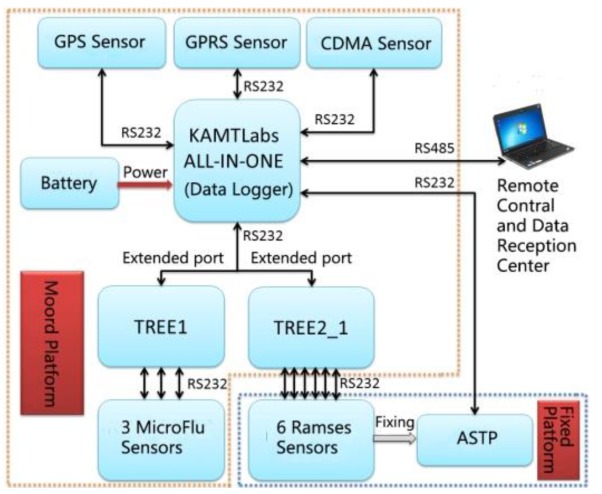
The diagram of the system design of the AFWCPOS (Automatic Stationary Water Color Parameters Observation System). The stationary platform is for spectra measurements, and the moored one is for the data logger and power supply. In addition, the automatic sun-tracking platform (ASTP) is an automatic sun-tracking platform. (GPS: Global Positioning System; GPRS: General Packet Radio Service; CDMA: Code Division Multiple Access; KAMTLabs: Labs of Kaner Application Marine Technology; TREE is a device of extended serial port)

**Figure 4:**
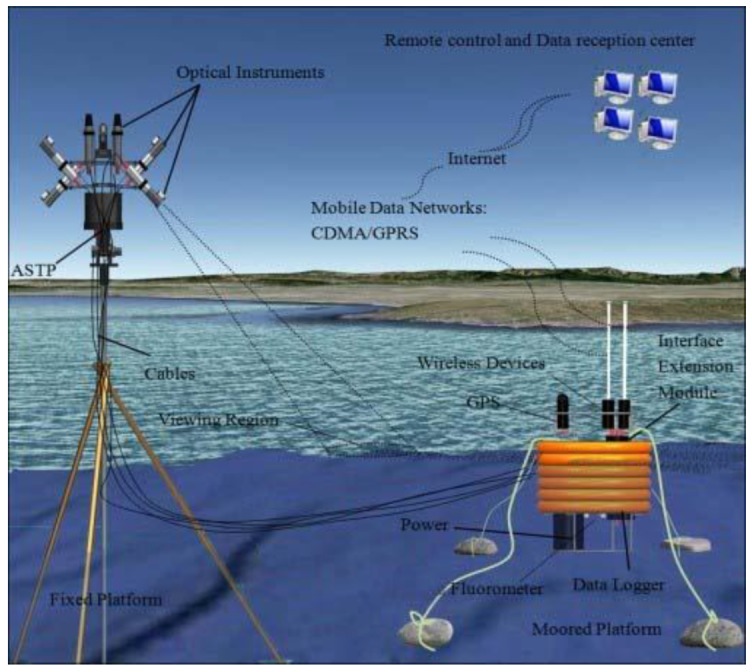
The deployment scheme for the AFWCPOS.

Figure 3 shows a diagram of the main AFWCPOS components. The AFWCPOS consists of two platforms, a fixed one to ensure that the water spectral data is measured under stable conditions, and the moored one for the wireless system, data-acquisition modules, and power supply. The deployment scheme is displayed in Figure 4. The core components of the AFWCPOS are an original design, including the platform for the unattended spectral collection, the automatic sun-tracking platform, the remote control and data processing system, etc.

### 3.1. Spectral Measurement System on the Stationary Platform

**Figure 5:**
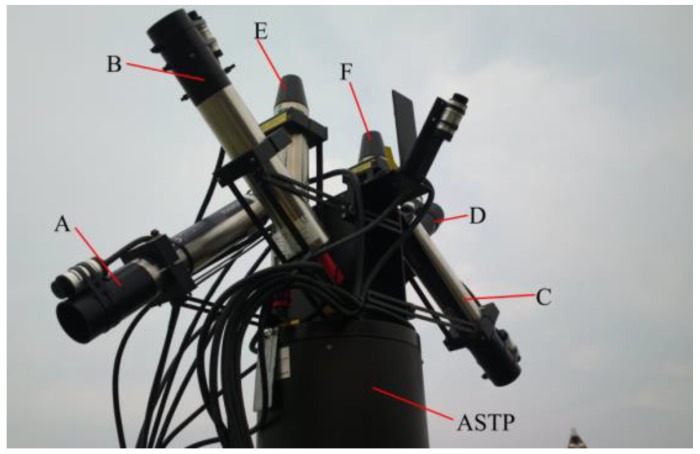
Six hyperspectral TriOS-RAMSES radiometers mounted on the automatic sun-tracking platform (ASTP) of the AFWCPOS, including four RAMSES-ARC sensors for radiance measurement (A, B, C, and D) and two RAMSES-ACC sensors for irradiance observation (E and F).

In this study, six laboratory-calibrated TriOS RAMSES hyperspectral spectroradiometers, two for irradiance at a view zenith angle of 0° and four for radiance at the view zenith angle of 135° or 45°, were mounted on the ASTP to ensure that the measurements were comparable (Figure 5). Six TriOS RAMSES sensors, which have been calibrated by producer, were installed on two sides of the automatic sun-tracking platform (ASTP). Three sensors on one side consist of two RAMSES-ARC radiometers (A and B), which are used for measuring the upwelling radiance (*L_u_*) at a nadir angle of 40° and the sky radiance (*L_sky_*) at a zenith angle of 40°, and one RAMSES-ACC (E), which is used for measuring the downwelling irradiance (Ed). The spectral range is from 350 nm to 950 nm, which contains the complete visible spectrum and parts of the ultraviolet and near-infrared. The optical system of the TriOS RAMSES-ARC sensor is composed of an optical fiber and a fused silica lens. The sensor has a spectral resolution of 10 nm with a 7° field of view. Trios RAMSES-ACC irradiance sensors use white cosine collectors made from fused silica, which are placed at the front of the instrument to diffuse the light into the optical fiber behind it. The output of the irradiance sensors is given as a power incident on a surface per wavelength. The sampling time interval is set by a remote controller, and eight measurements were recorded for each sample. In addition, the ASTP calculates the solar azimuth of the test site and adjusts the viewing geometry automatically according to the sun’s position.

### 3.2. Data Collection System on the Moored Platform

The autonomous system relies on wireless communication to transmit data and receive instructions to ensure that the whole system works properly. In our experiment, the optical instruments and the ASTP on the fixed platform were connected to a moored buoy by cables. The primary equipment on the moored platform consists of a data logger, two wireless devices, a GPS device, two interface extension modules, and the system power supply. In addition, three WET Labs ECO fluorometers are mounted on the moored platform to collect the Chl-a, NTU, and CDOM data. This was the first time that WETLabs ECO fluorimeters had been used for a field experiment after factory calibration, and the data are reliable. According to technical documentation of WETLabs, Chl-a concentration, Turbidity, and CDOM can be derived using the equation:

Chl-a (μg/L), Turbidity (NTU), and CDOM (ppb) = Scale Factor * (Output − Dark Counts)

where Dark Count Signal is the output of the meter in clean water with black tape over the detector, and Output is signal recorded by fluorimeter. Scale Factor is determined using the following equation: SF = x ÷ (output − dark counts), where x is the concentration of the solution used during instrument characterization for Chl-a and CDOM, and the value of a Formazin concentration for turbidity. These parameters are shown in Table 1. In addition, the maximum output and resolution (standard deviation of 1 min of collected data) are also been shown in Table 1.

**Figure 6:**
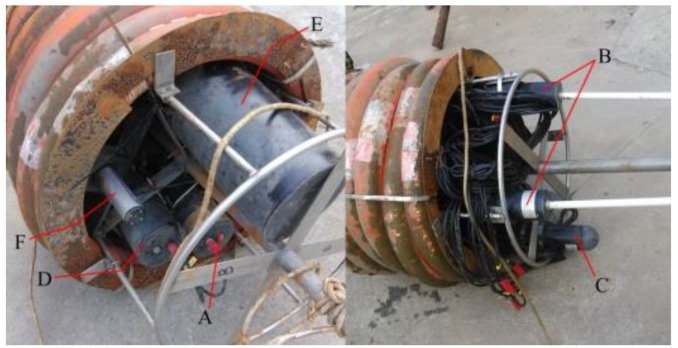
The moored platform consists of: (**A**) a data logger, (**B**) two wireless devices, (**C**) a GPS device, (**D**) two interface extension modules, (**E**) the system power supply, and (**F**) fluorometers.

The data logger plays a key role in controlling the system, and it is primarily responsible for system configuration, supervision, data collection, storage, transmission, and responding to user’s commands, among other functions. The data logger can receive and store a variety of signals that TriOS, GPS, and other wireless devices spread. As shown in Figure 6, the data logger connects to the pre-configured instruments, including the wireless device, GPS device, and interface extension module. The battery compartment is responsible for providing power to the data logger and all instruments. The wireless device is used for data transmission. The GPS device provides location and time information in all weather conditions. Measurement sequences are performed with user-definable intervals and frequencies, and the integration time of the TriOS RAMSES sensors varies automatically between 4 ms and 8192 ms, depending on the brightness of the target.

## 4. Applications of the AFWCPOS in Shallow Waters

### 4.1. The Experiment in the Poyang Lake

**Figure 7:**
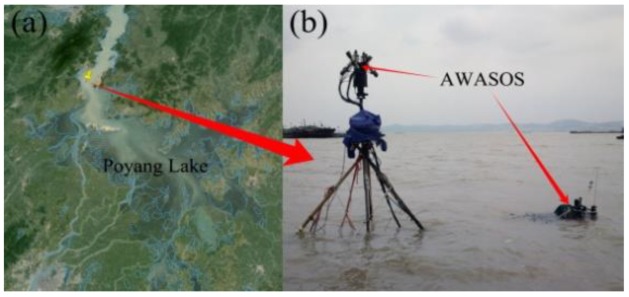
(**a**) The deployment site (29°26.75′ N, 116°3.1′ E) and (**b**) the real scene in Poyang Lake.

Poyang Lake, the largest freshwater lake in China, is located in the mid-lower region of the Yangtze River in southeastern China (28°22′~ 29°45′ N and 115°47′~ 116°45′ E) (Figure 7). Influenced by subtropical monsoons, the inundation area of Poyang Lake varies from approximately 3000 km^2^ during the wet season to approximately 1000 km^2^ during the dry season [19]. Existing studies primarily focus on water quality monitoring from MODIS [1], Landsat TM/ETM+ [20], HJ-1 CCD [21], or GF-1 WFV [22] at various spatial and temporal scales. Although the long-term (inter-annual and seasonal) dynamics of the lake, including the increased SPM (suspended particulate matter) concentration and declining water quality, have been well-documented [1,23,24,25], stationary high-frequency spectral and water constituents concentration observations, especially Chl-a, NTU, and CDOM measurements, are rare and urgently needed.

The AFWCPOS was deployed in the Poyang Lake (29°26.75′ N, 116°3.1′ E) from 20 to 28 July 2013 (see Figure 7). The deployment site is near Xingzi County, Jiujiang City, Jiangxi Province, China. The water depth is approximately 3 m. The spectral data were collected from 9:00 to 18:00, when the weather was fine and cloudless, average wind speed less than 3 m/s. Due to the lack of an automatic cleaning unit for moored optical sensors, we manually cleaned them every day against the fouling problem. Besides, the SVC HR-1024 field-portable spectroradiometer was employed to collect *Rrs* simultaneously on a ship, which had a viewing direction of 50° from the nadir and 135° from the sun to minimize the effects of sun glint and to avoid instrument shading problems, according to the recommended protocols for optical measurements [13].

### 4.2. Validation and Cross Comparison of Spectral Data from the AFWCPOS and Conventional Approach

To assess the performance of the AFWCPOS proposed in this paper, the remote sensing reflectance dataset *R*_rsAFWCPOS_ were collected by the AFWCPOS. Meanwhile, the simultaneous dataset *R*_rsSVC_ was collected by an SVC HR-1024 field-portable spectroradiometer on a ship.

**Figure 8:**
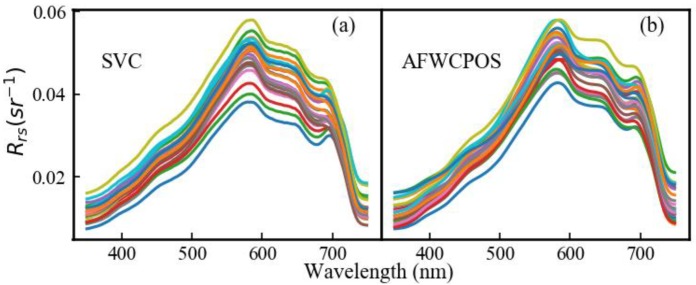
The remote sensing reflectance (Rrs(sr-1)) in Poyang Lake (29°26.75′ N, 116°3.1′ E) from measurements made by (**a**) the SVC (Spectra Vista Corporation) HR1024 and (**b**) the Automatic Stationary Water Color Parameters Observation System (AFWCOS).

The qualitative comparison of *R*_rsAFWCPOS_ and *R*_rsSVC_ is shown in Figure 8. The spectral profile of two data sets was highly similar and was consistent with a previous study [22]. More specifically, three reflectance peaks at approximately 580 nm, 650 nm, and 700 nm were also found, as shown in Figure 8. The reflectance peak at approximately 580 nm was due to the decreased absorbance of phytoplankton below 580 nm, together with the dramatically increased absorbance of pure water above 580 nm. The evident absorbance peaks of phycocyanin at approximately 620 nm and phytoplankton or mineral particles at approximately 675 nm caused the reflectance peak at approximately 650 nm. The reflectance peak at approximately 700 nm was due to the obvious absorbance peak of phytoplankton or mineral particles at approximately 675 nm and the dramatically increased absorbance of pure water above 700 nm. These results were similar to the results of a previous study in Poyang Lake [21].

**Figure 9:**
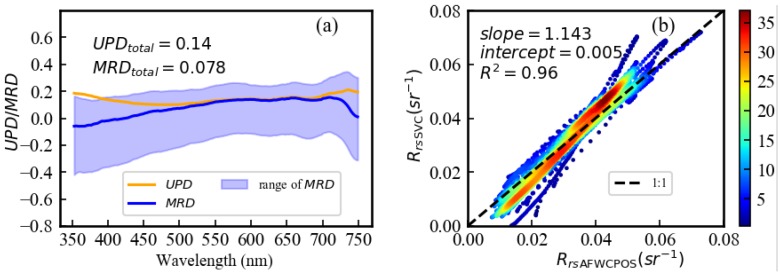
(**a**) The UPD (unbiased percent difference) and MRD (mean relative difference) as a function of wavelength for *R*_rsSVC_ and *R*_rsAFWCPOS_. (**b**) Comparison of *R*_rs_ dataset from the AFWCPOS (*R*_rsAFWCPOS_(λ)) and simultaneous manual measurements (*R*_rs__SVC_(λ)) provided by an SVC HR-1024 field-portable spectroradiometer at Poyang Lake.

For the quantitative validation of spectrum collection from AFWCPOS, the unbiased percent difference (UPD, Equation (3)), coefficient of determination (*R*^2^), and the mean relative difference (MRD, Equation (4)), were derived from *R*_rsAFWCPOS_ and *R*_rsSVC_.

(3) $$UPD=\frac{1}{N}\overset{N}{\underset{i=1}{\sum }}\frac{\left|\left(x_{i}-y_{i}\right)\right|}{0.5*\left(x_{i}+y_{i}\right)}$$

(4) $$MRD=\frac{1}{N}\overset{N}{\underset{i=1}{\sum }}\frac{\left(x_{i}-y_{i}\right)}{x_{i}}$$

where $x_{i}$ is the *R*_rsSVC_ value at the data matchup i, and $y_{i}$ is the corresponding *R*_rsAFWCPOS_. UPD is used to provide an absolute difference between two data sets, and MRD is used to provide a relative difference [26]. From Figure 9a, the total UPD and total MRD between *R*_rsSVC_ and *R*_rsAFWCPOS_ were 0.14 and 0.078. The UPD and MRD were lower than 20% and 10%, respectively, for the entire wavelength. The range of MRD is from –0.4 to 0.2, and the MRD and the UPD are stable, especially from 450 nm to 700 nm. The combination of weak reflectance signal and stable noise may cause relatively large error below 450 nm and above 700 nm; thus, it was demonstrated that the random error is not critical for the accuracy of *R*_rsAFWCPOS_. A correlation analysis between the automatic spectral data (*R*_rsAFWCPOS_(λ)) and the manual spectral data (*R*_rsSVC_(λ)) was performed, and results proved significant consistency between these two datasets, with the regression slope near 1 and coefficient of determination, *R*^2^, of 0.96 (Figure 9b).

However, there are still some disagreements between the *R*_rs__AFWCPOS_ and the *R*_rs__SVC_ measurements based on the above analysis. Possible causes of such inconsistency may include: first, discrepancies of instrument parameters among varied sensors (FOVs: field of view; SNRs: ratio of signal and noise) and measurement conditions (such as measuring time); and most importantly, the optical sensors of the SVC are manually operated to take all measurements without following the rigorous viewing geometry, so there is uncertainty factor that Fresnel reflectance to corrupt *R*_rs_ is collected with two spectrometer systems. Besides, the differences in the instruments’ calibrations also affect the results of the data processing.

### 4.3. Applications and Potential of the AFWCPOS in Routine Water Quality Monitoring

#### 4.3.1. Validation of the AFWCPOS-Measured Chl-a Concentration

**Figure 10:**
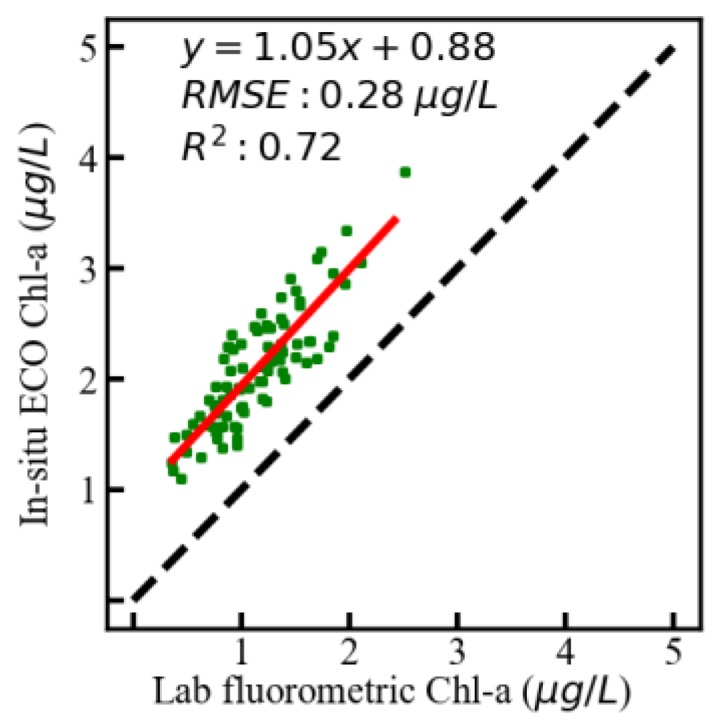
Regression analysis between lab fluorometric and in-situ ECO (Environmental Characterization Optics) chlorophyll-a (Chl-a) concentration.

Chl-a concentration from ECO measurement was compared with data collected using a lab-based fluorometric analysis method. After obtaining water samples, the chlorophyll samples were filtered through 0.45 μm Whatman cellulose acetate membranes. Then, the filters were soaked with ethanol (90%) at 0 °C for 24 h for extracting Chl-a pigments. The concentration of chlorophyll (Chl) (μg/L) was determined by measuring the extracted pigment samples using an RF-5301 Fluorescent Spectrophotometer (Shimadzu, Kyoto, Japan), which has been calibrated by the Chl-a standards manufactured by Sigma Chemical Co. (St. Louis, MO, USA). In the experiment, the exciting and fluorescent wavelengths were 432 and 667 nm, respectively. Finally, the in-situ ECO Chl-a was validated using the regression analysis method (Figure 10).

Comparing between lab fluorometric and in-situ ECO Chl-a, a linear relationship showed a high level of consistency, with a slope of 1.05, the mean relative squared error of 0.28 μg/L, and the determining coefficient of 0.72. 

#### 4.3.2. Short-Term Features of Chl-a, Turbidity, and CDOM at Poyang Lake

**Figure 11:**
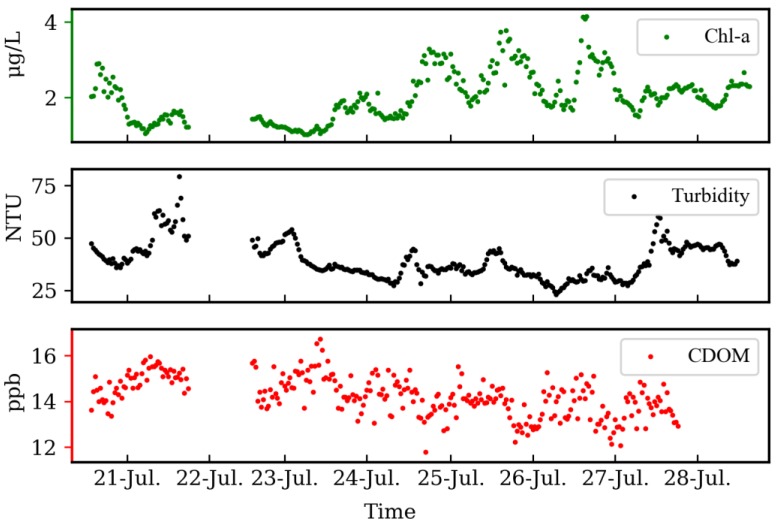
The concentration of Chl-a, Turbidity, and CDOM (Colored Dissolved Organic Matter) in the deployment site (Figure 7) from 20 July 2013, to 28 July 2013.

Chl-a, turbidity, and CDOM data collected by three ECO fluorometers in Poyang Lake from 20 to 28 July 2013 are presented in Figure 11. The cause of data missing was instrument maintenance. It demonstrated significant diurnal and inter-diurnal variations in Poyang Lake, but the three constituents were always in the state of dynamic balance with distinct different periodic variations. In terms of Chl-a, obvious regularity could be observed with concentration reaching bottom about five or six in the morning and hitting peak every afternoon; this phenomenon might be related to the biological activity of aquatic plants and Phytoplankton, being affected by temperature and sunshine. By contrast, although the regularity was not relatively obvious, the trend of turbidity and CDOM concentration was still being observed.

**Figure 12:**
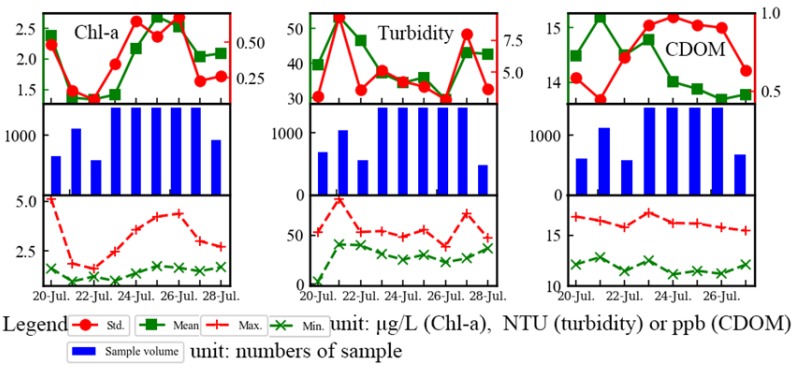
Statistics of Chl-a, turbidity, and CDOM in the deployment site (Figure 7) from 20 July 2013, to 28 July 2013.

Several statistics of Chl-a, turbidity, and CDOM concentration are shown in Figure 12. The average, standard deviation, maximum, and minimum were represented by square symbols, dot symbols, plus sign, and multiplication sign, respectively, and the blue bar indicates data volume collected every day. Significant diurnal and inter-diurnal variations could be observed. By comparing the statistics on 20 July, 21 July, 22 July, and 28 July with other days, the discrepancies were obvious, which might be caused by interrupted data collection. Therefore, an uninterrupted observation by using automated instruments could help to accurately understand water information in the high-dynamic lake, such as Poyang Lake.

### 4.4. Remote Sensing Application of AFWCPOS

To validate the effectiveness of AFWCPOS in satellite remote sensing monitoring, collected data were used to establish empirical relationships between water quality parameters (Chl-a, turbidity, and CDOM) and remote sensing reflectance, respectively. We chose Landsat 8 OLI image as remote sensing data in this experiment because of its high quality and high spatial resolution in abroad applications.

Before establishing a model, simulated Landsat 8 OLI specific band remote sensing reflectance (*Rrs*(band)) was concluded from integrating AFWCPOS spectrum data (*Rrs*(λ)) and relative spectral response (*RSR*(λ)) according to Equation (6).

(5) $$R_{rs}\left(band\right)=\frac{\int R_{rs}\left(\lambda \right)RSR\left(\lambda \right)d\lambda }{\int RSR\left(\lambda \right)d\lambda }$$

**Figure 13:**
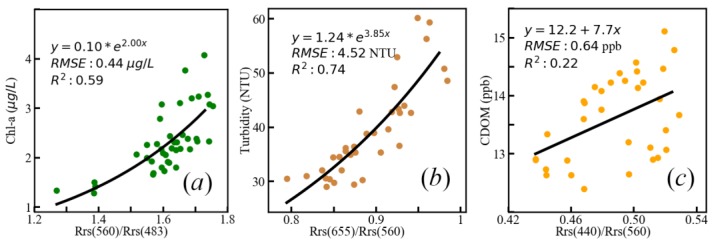
Regression model of water quality parameters with the ratio of simulated Landsat 8 OLI (Operational Land Imager) remote sensing reflectance. (**a**): Chl-a, (**b**) turbidity, (**c**) CDOM.

Thus, the retrieving algorithms of three water quality parameters were built from bands ratio of remote sensing reflectance (Figure 13), from which the retrieving models of Chl-a, turbidity, and CDOM were:

(6) $$\mathrm{Chl}\text{-}a:\;y=0.1*e^{2*\frac{Rrs\left(560\right)}{Rrs\left(483\right)}}$$

(7) $$\mathrm{Turbidity}:\;y=1.24*e^{3.85*\frac{Rrs\left(655\right)}{Rrs\left(560\right)}}$$

(8) $$\mathrm{CDOM}:\;y=7.7*\frac{Rrs\left(440\right)}{Rrs\left(560\right)}+12.2$$

Significant relationships between Chl-a and turbidity and specific band ratios were observed with the determining coefficients of 0.59 and 0.74, and the root mean squared errors of 0.44 μg/L and 4.52 NTU, respectively. For CDOM retrieval algorithm, the relative feature of distribution could be mapped based on the above model (Equation (9)), even though lower *R*^2^ caused by the discrete degree of in-situ CDOM was obtained from AFWCPOS (see Figure 11) as its significant periodic characters, shown in Figure 10. Therefore, these models should be considered acceptable and valid.

**Figure 14:**
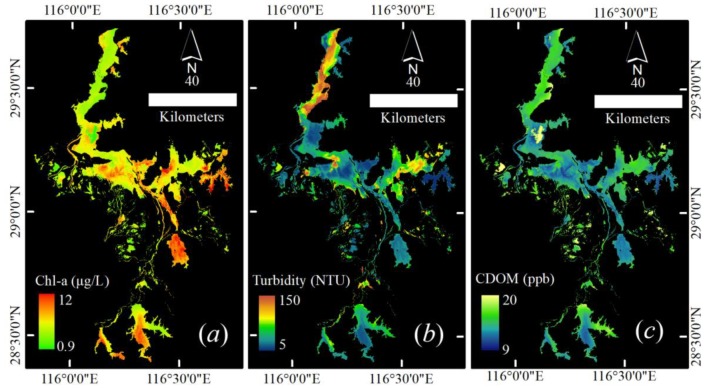
Water quality parameters derived from OLI/Landsat 8 on 5 October 2013, for Poyang lake. (**a**) Chl-a, (**b**) turbidity, (**c**) CDOM.

Three retrieving models were applied in a specific case for mapping spatial distribution of Chl-a, turbidity, and CDOM in Poyang lake derived from OLI/Landsat 8 on 5 October 2013 (Figure 14).

**Figure 15:**
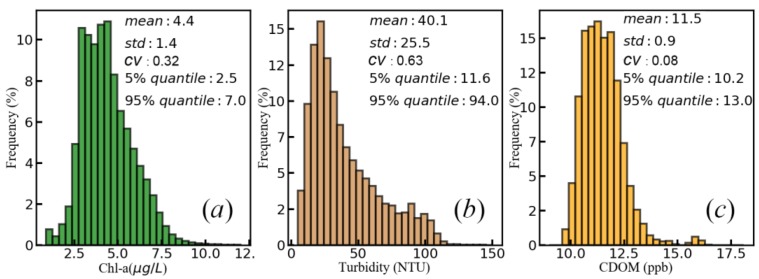
The histograms and several statistic indexes of retrieval results.

The result revealed a large spatial variation between the northern and southern lake areas. The northern area was dominated by the high turbidity, which was induced by intense sand dredging activities and hydrodynamic force affected by the Yangtze River in the narrow water channel of the northern lake [1]. In the southern area, a high concentration of Chl-a was observed, which was induced by rich nutrition from southeastern rivers (see Figure 7a) and better light conditions under the water because of relatively lower turbidity. In addition, limited water exchange was another reason causing a high concentration of Chl-a in southeastern sub-lakes [27]. A similar spatial distribution between CDOM and turbidity illustrates the sediment carrying main CDOM in Poyang lake. However, Chl-a degradation in lower turbidity area would increase CDOM level, which led to relatively lower spatial heterogeneity than turbidity and Chl-a (comparison between CVs, coefficients of variation, of water quality parameters in Figure 15).

In Figure 15, the other statistic indexes of retrieval results of Chl-a, turbidity, and CDOM recorded more detailed information. Despite larger ranges of values, 90% of areas ranged from 2.5 to 7 μg/L for Chl-a, from 11.6 to 94 NTU for turbidity, from 10.2 to 13 ppb for CDOM, respectively. Because of extremely high spatial-heterogeneity, the histogram of turbidity showed a more skewed distribution. CDOM, by contrast, had a normal distribution, relatively. These statistic features reflected the water quality level in Poyang lake.

The results of this case were similar to previous researches [1,27], pointing out that the spectrum and water quality parameters obtained from AFWCPOS could be used in water color remote sensing study.

## 5. Conclusions

The Automatic Stationary Water Color Parameters Observation System (AFWCPOS) proposed in this paper is an operational system for routine water quality monitoring, with the advantages of automatic data acquisition, storage, and wireless transmission, and would thus greatly reduce uncertainties of artificial operation from conventional methods. Applications in Poyang Lake showed the capacity of AFWCPOS to obtain time series, high-frequency, and high accuracy data of water spectral and water color parameters. Meanwhile, AFWCPOS could also obtain reliable data for water color remote sensing. Chl-a, turbidity, and CDOM were mapped from Landsat 8 OLI image and in-situ data obtained by AFWCPOS. The spatial distributions were similar to previous researches. Compared with the conventional method, the AFWCPOS improved the temporal scale significantly at a low cost. In addition, it could provide an integrated solution for those optical sensor owners. Further research on the system would focus on reducing the cost of the system by using spectral sensors (self-developed or commercial sensors) based on high-speed multiplexers, and methods to eliminate errors of different optical sensors. Some effective measures should be taken to ensure that the optical sensors are not contaminated by atmospheric ash and that the electrical modules are kept away from the water. Therefore, onboard calibration instruments and methods will be added in the future. In addition, we believe that more field sensors would allow more data to be collected without interference for dynamic water quality monitoring because of the development of the data logger.

## Figures and Tables

**Table 1:** The calibration coefficients and characterization of ECO (Environmental Characterization Optics) fluorometers.

Item	Scale Factor	Dark Counts	Maximum Output	Resolution
Chl-a ^1^ (μg/L)	80 μg/L/V	0.024 V	5 V	0.5 mV
Turbidity (NTU)	201 NTU/V	0.019 V	4.99 V	0.2 mV
CDOM ^2^ (ppb)	100 ppb/V	0.019 V	4.97 v	0.5 mV

^1^ Chlorophyll-a. ^2^ Colored dissolved organic matter.
